# Bilateral Acute Proptosis With Papilledema and Sub-retinal Fluid As Initial Manifestations of Acute Lymphoblastic Leukemia in a Child

**DOI:** 10.7759/cureus.64203

**Published:** 2024-07-09

**Authors:** Elpida Kollia, Susmito Biswas, Bhamy Hariprasad Shenoy

**Affiliations:** 1 Paediatric Ophthalmology, Manchester Royal Eye Hospital, Manchester, GBR

**Keywords:** papilledema, pediatric hematology-oncology, sub-retinal fluid, uncommon leukemia presentations, acute lymphoblastic leukemia (all)

## Abstract

A five-year-old boy presented with bilateral acute proptosis, papilledema, and sub-retinal fluid. Notably, choroidal thickening exceeded 600 microns. These ocular findings were the initial manifestations of acute lymphoblastic leukemia. This case underscores the importance of recognizing uncommon ocular presentations in pediatric leukemia for timely diagnosis and management.

## Introduction

Acute lymphoblastic leukemia (ALL) is the most common pediatric malignancy, characterized by the proliferation of immature lymphoid cells in the bone marrow and peripheral blood. The worldwide incidence of ALL is approximately 3.5-4.0 per 100,000 children annually, with a peak incidence between two and five years of age. Typically, ALL presents with systemic symptoms such as fatigue, pallor, fever, and a tendency to bleed or bruise easily. These symptoms result from bone marrow infiltration and the subsequent reduction in normal blood cells. However, extramedullary involvement in ALL, particularly with ocular manifestations, is rare and can present significant diagnostic challenges [1].

Ocular manifestations of ALL can be diverse, involving various structures of the eye and orbit. They may include retinal hemorrhages, papilledema, optic nerve infiltration, and proptosis [2,3]. Such manifestations are often overlooked because they are not commonly associated with leukemia and may lead to delayed diagnosis and treatment. Recognizing these atypical presentations is critical for timely intervention, which can significantly improve the prognosis and reduce the risk of severe complications.

This case report describes a five-year-old boy who presented with unusual ocular symptoms as the initial manifestation of ALL. The early identification of these symptoms led to a prompt diagnosis and the initiation of appropriate treatment, highlighting the importance of awareness and consideration of leukemia in the differential diagnosis of pediatric patients with ocular abnormalities. This case emphasizes the need for a thorough clinical evaluation and collaboration with specialists to ensure accurate diagnosis and effective management of such rare presentations.

## Case presentation

A five-year-old previously healthy boy presented with bilateral acute proptosis, papilledema, and sub-retinal fluid. The uncorrected visual acuity was notably reduced, measured at 0.84 in the right eye and 0.2 in the left eye (LogMAR), with no pinhole improvement and no refractive error identified. Clinical examination revealed bilateral exophthalmos with unrestricted ocular motility and no strabismus, along with a right relative afferent pupillary defect. The anterior segments appeared unremarkable, with normal intraocular pressures noted during the examination. Fundoscopy findings included intra-retinal hemorrhages, vessel tortuosity, papilledema, and bilateral sub-retinal fluid.

**Figure 1:**
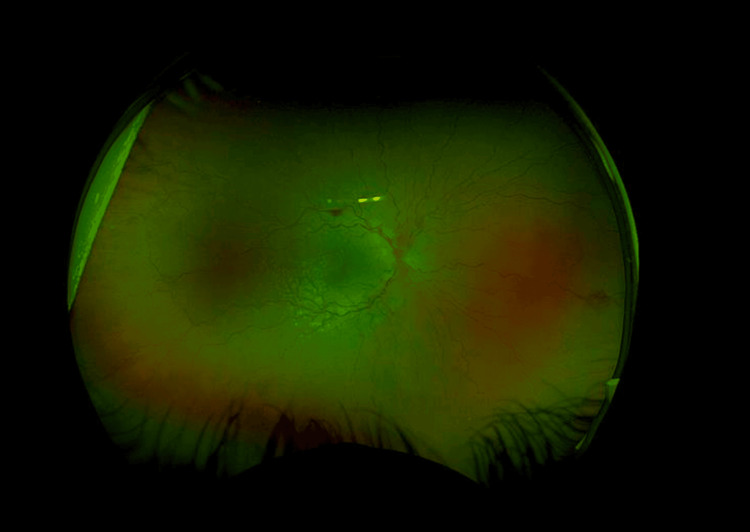
Colored retinal image of the right eye illustrating optic disc swelling with vessel tortuosity and retinal hemorrhages

**Figure 2:**
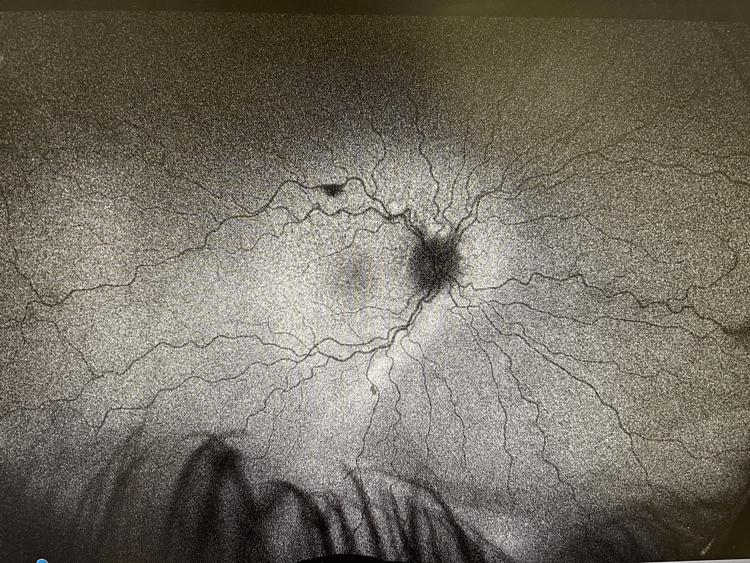
Autofluorescence of the right eye

**Figure 3:**
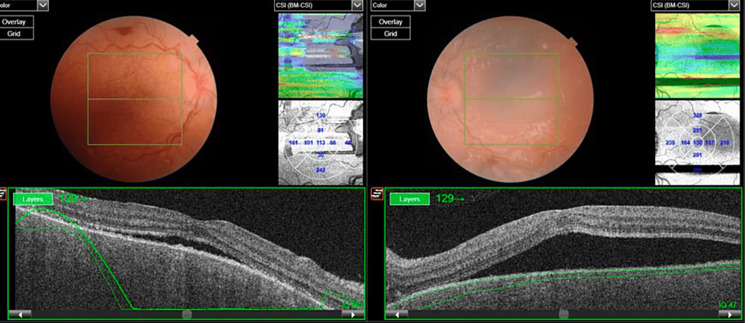
OCT-macula of both eyes highlighting excessive sub-retinal fluid and choroidal thickening OCT: optical coherence tomography

**Figure 4:**
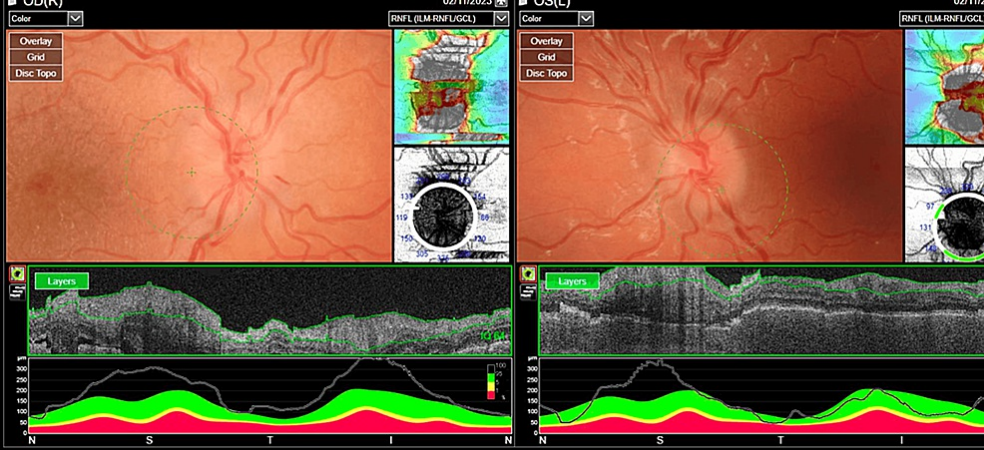
OCT-RNFL upon the first visit OCT: optical coherence tomography, RNFL: retinal nerve fiber layer

Retinal imaging revealed substantial choroidal thickening, surpassing 600 microns (637 µm), prompting consideration of an infiltrative process (Figures 1-4). To ensure the anonymity of the patient, images have not been included. The differential diagnosis included various etiologies, such as infectious, inflammatory, and neoplastic conditions. Space-occupying lesions, including tumors, were considered given the bilateral proptosis and papilledema. However, the absence of findings suggestive of infection or inflammation, coupled with the presence of atypical lymphoblasts in cerebrospinal fluid, pointed towards a hematologic malignancy.

Non-ophthalmic investigations included initial MRI and CT scans of the brain and orbits; initially unremarkable but adjusted MRI sequences confirmed optic nerve infiltration upon further review by the neuroradiology team. A lumbar puncture revealed elevated intracranial pressure and atypical lymphoblasts in cerebrospinal fluid. A complete blood count demonstrated leucocytosis with a predominance of lymphoblasts, confirming B-cell precursor ALL.

Laboratory investigations, such as cytogenetics, indicated Philadelphia-chromosome-positive ALL, highlighting the genetic profile of the leukemia. The patient's CNS status was closely monitored and classified as CNS status 3. Additionally, echocardiography showed no effusion with preserved cardiac function (EF 48%).

**Figure 5:**
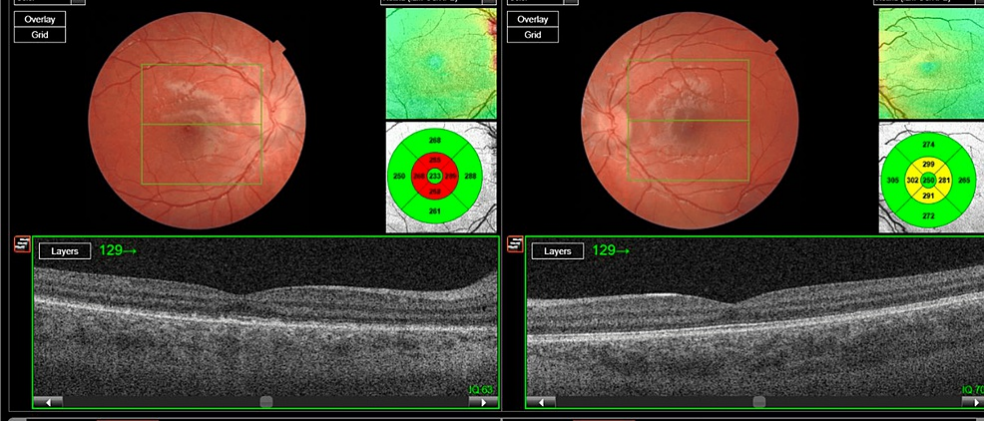
OCT-macula one month following treatment initiation OCT: optical coherence tomography

Prompt initiation of standardized ALL chemotherapy protocols led to gradual improvement in ophthalmic symptoms. Following induction chemotherapy, the patient showed significant recovery, with consolidation and maintenance phases contributing to continued progress. One month post-treatment initiation, visual acuity improved to 0.14 in the right eye and 0.04 in the left eye (LogMAR), accompanied by resolution of sub-retinal fluid (Figure 5).

## Discussion

The presented case underscores the rarity of ocular symptoms as the primary manifestation of pediatric ALL. While ALL commonly presents with systemic symptoms such as fatigue and bruising, extramedullary involvement, especially in the orbit, is relatively uncommon [2-4]. Ocular manifestations as the initial presentation of leukemia are exceedingly rare and have not been extensively reported in the literature.

Early recognition of ocular manifestations of leukemia is paramount for the timely diagnosis and initiation of treatment [5]. Prompt management with chemotherapy and systemic therapy can prevent further progression of the disease and improve overall outcomes. Additionally, close monitoring of ocular symptoms during and after treatment is essential to assess treatment response and detect any relapse or recurrence.

The literature includes several cases documenting ocular presentations of leukemia, such as orbital involvement, optic nerve infiltration, and bilateral optic disc swelling [6-10]. Reports have described cases of ALL and acute myeloid leukemia presenting with proptosis, papilledema, and optic disc swelling in both pediatric and adult populations. These reports contribute to our understanding of the diverse ocular manifestations of leukemia and underscore the importance of considering leukemia in the differential diagnosis of pediatric patients with ocular symptoms.

Moreover, collaboration with neuroradiology for accurate imaging is crucial. In this case, an adjusted MRI sequence led to the detection of optic nerve infiltration, which was pivotal for diagnosis. This case highlights the importance of a multidisciplinary approach to managing complex presentations and ensuring comprehensive care for pediatric patients.

## Conclusions

This case highlights the rare occurrence of ocular symptoms, such as bilateral acute proptosis and papilledema, as the initial presentation of pediatric ALL. Early recognition of these unusual manifestations is crucial for timely diagnosis and treatment, leading to significant improvement in ocular symptoms and overall patient outcomes.

Prompt initiation of chemotherapy, collaboration with neuroradiology for accurate imaging, and continuous monitoring of ocular symptoms are essential for effective management. This case contributes to the understanding of diverse presentations of leukemia and underscores the importance of considering hematologic malignancies in pediatric patients with atypical ocular symptoms.
